# The Church, Practitioners, and the Marriage of Syphilitics

**Published:** 1919-06-28

**Authors:** 


### The Hospital

The Workers' Newspaper of Administrative Medicine and Institutional Life, Administration, National Insurance and Health.

No. 1725, Vol. LXVI. SATURDAY, JUNE 28, 1919. PRICE TWOPENCE

### THE CHURCH, PRACTITIONERS, AND THE MARRIAGE OF SYPHILITICS.

A correspondent has written us a letter which will be found on p. 331 of the present issue. *Prima facie* it would appear that such action on the part of a priest, as our correspondent describes, is infamous, but the matter is not so simple as it appears. Few social or legal problems are. Has a priest any power to refuse to perform the marriage ceremony over a couple of parishioners whose banns have been duly proclaimed without objection being taken? We are not sufficiently conversant with ecclesiastical law to express an opinion on the point, but we greatly doubt whether a parish priest has any such power, though no doubt he may refuse to solemnize a marriage by licence, which is permissive, but not obligatory.

Supposing, however, that the priest has the power to refuse to perform the ceremony, would he be justified in refusing on the ground mentioned if he became acquainted with the fact—if it is a fact—merely by hearsay? A refusal to perform the marriage ceremony is a serious matter, and to refuse on the ground stated is a very serious matter, not to be lightly undertaken. A prudent priest would scarcely be justified in refusing even if he had a written and signed statement from an informant in a position to know. He would want a statutory declaration or an affidavit. It would be difficult for him to refuse without stating the grounds of his refusal, and if he did state them, even on the authority of an affidavit, he would lay himself open to an action for slander. But is our correspondent himself without blame? If the marriage was by banns, it was open to him to forbid the banns, and thus throw upon the priest a duty to make enquiry, and to hear and determine in a formal manner whether the reason alleged was a just cause or impediment to the marriage. If the marriage was by licence, he could have given information to the Vicar-General or to Surrogate, and thus have unburdened his conscience and thrown the responsibility upon the proper ecclesiastical authority. If he shrank from taking this step, it scarcely lies with him to find fault with the priest for shrinking from a still more invidious action.

Dr. Hawthorne advances certain considerations, which it appears he would call claims, against the practice advocated in The Hospital. They are sufficiently obvious, and were, of course, present in the mind of the writer of the article, but his remedy is a strange one. Professional secrecy must not be violated, but every prospective spouse must require of his or her partner a certificate of health. But suppose the prospective partner suffer from venereal disease? In that case a certificate of health would probably not be obtainable and whatever certificate was obtained must state the nature of the disqualifying disease. If it did not, enquiry would certainly be made, and if the desired information were not forthcoming, the marriage would certainly proceed. If it were revealed, what is become of professional secrecy? The plain fact is that the existence of venereal disease cannot be revealed without breach of professional secrecy, and whether this is done in conformity with an Act of Parliament or in response to private enquiry or from a sense of higher duty makes no difference to the fact that secrecy is violated. Dr. Hawthorne's method is a method by which secrecy is violated while a pretence is made that it is not violated, and like most pretences, it is ineffectual and could not increase our respect for the practice. In respect to the violation of secrecy, there is not much difference between saying that a man suffers from syphilis, and refusing to say that he does not suffer from it.

